# Comedo-like openings and milia-like cysts in a pigmented basal cell carcinoma: A dermoscopic pitfall

**DOI:** 10.1016/j.jdcr.2026.07.061

**Published:** 2026-08-07

**Authors:** Bruno Simão, Raisa Dttmann

**Affiliations:** a Santa Casa de Sao Paulo School of Medical Sciences, São Paulo, Brazil; b Private Practice, Dermatopathology, São Paulo, Brazil

**Keywords:** basal cell carcinoma, comedo-like openings, dermoscopy, seborrheic keratosis, ultraviolet-induced fluorescence dermoscopy

## Clinical presentation

A 64-year-old man with Fitzpatrick skin phototype IV and a history of nodular basal cell carcinoma of the scalp presented with an asymptomatic facial lesion that had enlarged over 1 year. Examination showed a well-circumscribed 5.5 × 4.0-mm hyperpigmented papule on the right temple (Fig 1).

**Fig 1:**
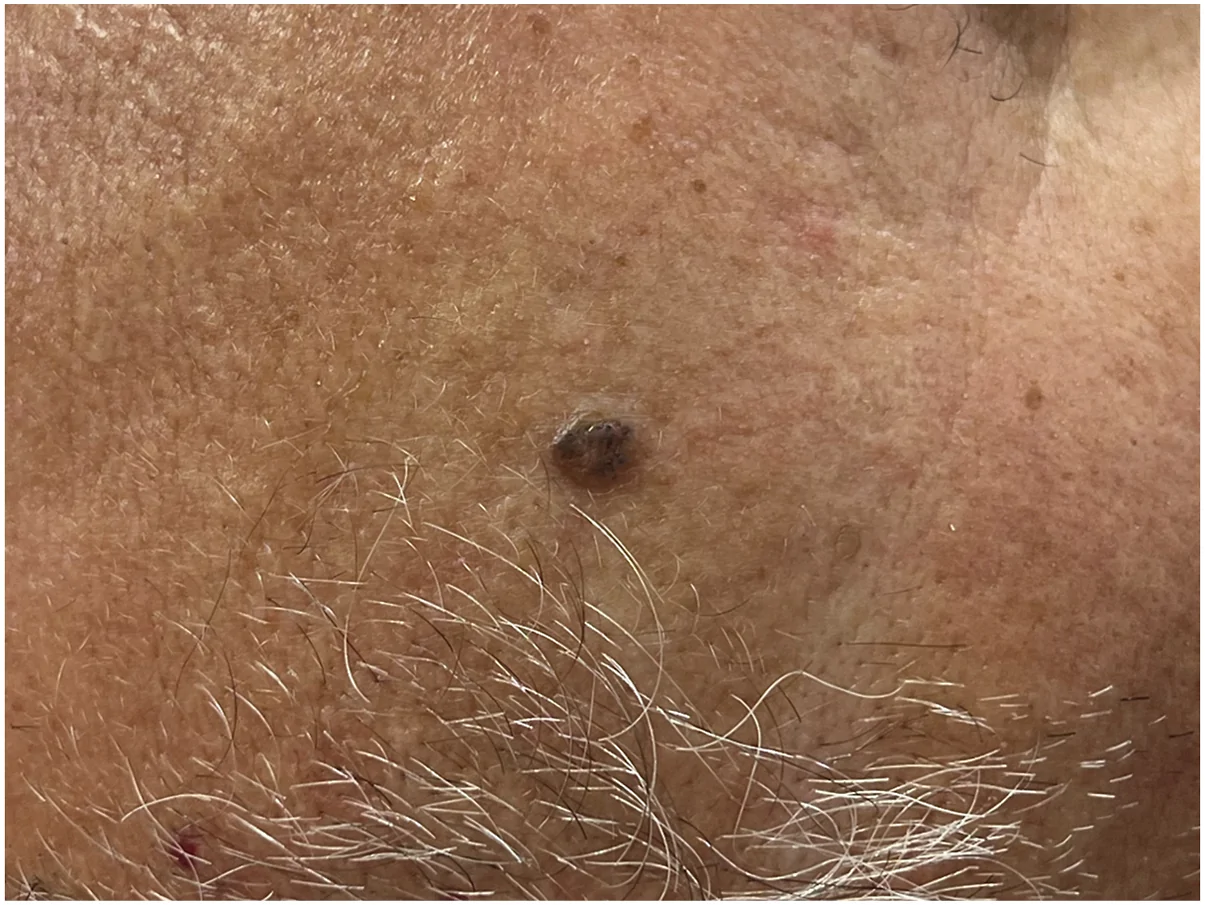
Clinical photograph showing a well-circumscribed hyperpigmented papule on the right temple.

## Dermoscopic appearance

Dermoscopic examination with nonpolarized and polarized light revealed structureless brown pigmentation, blue-gray ovoid nests, focused tortuous vessels, and comedo-like openings (Fig 2). Ultraviolet-induced fluorescence dermoscopy showed a dark silhouette of the lesion and multiple blue-white fluorescent clods corresponding to keratin-filled comedo-like openings (Fig 3). Milia-like cysts were identified only under nonpolarized light and showed no fluorescent signal.

**Fig 2:**
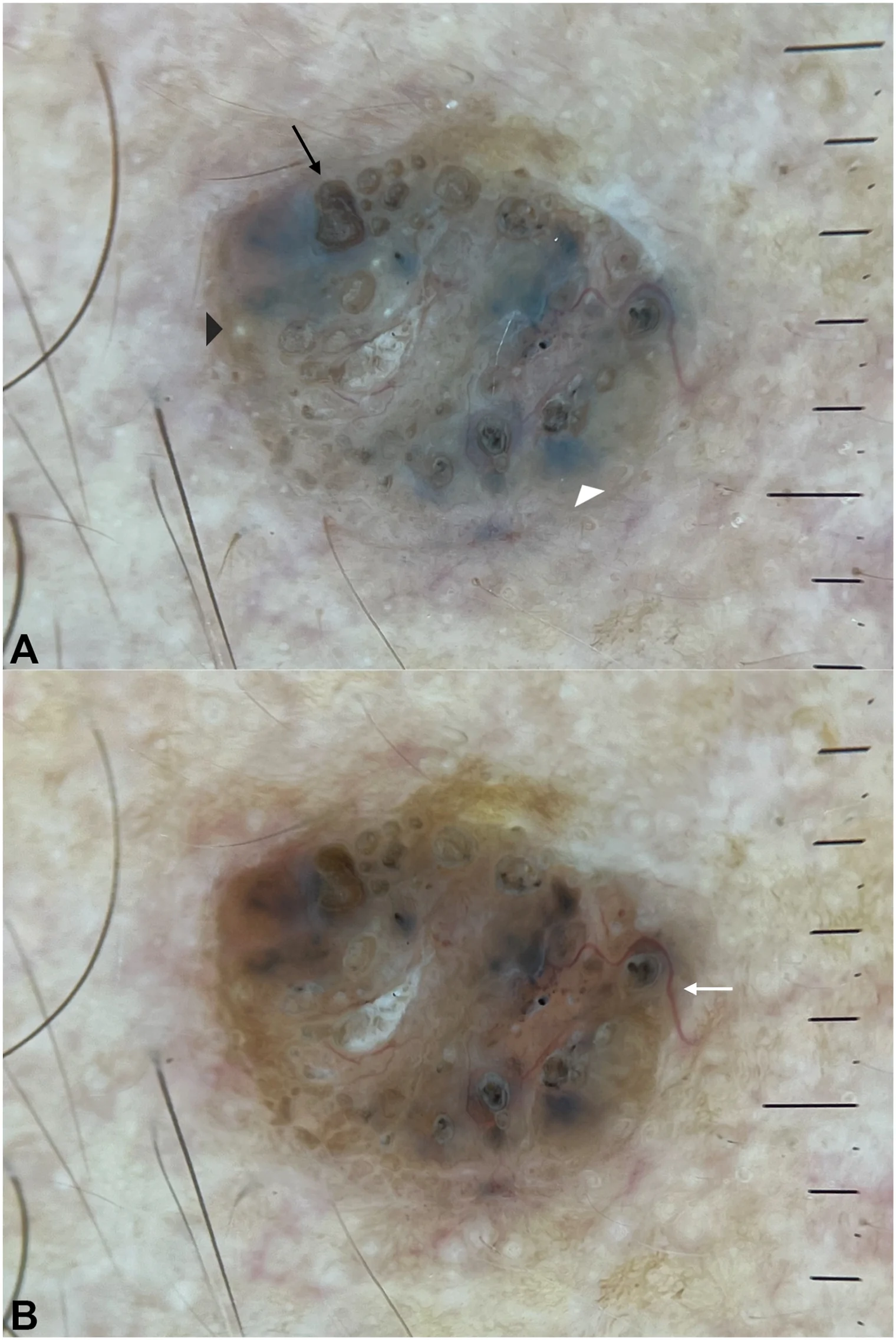
**A,** Nonpolarized dermoscopic image showing structureless brown pigmentation with blue-gray ovoid nests (*white arrowhead*), comedo-like openings (*black arrow*) and milia-like cysts (*black arrowhead*). Original magnification ×10. **B,** Polarized dermoscopic image revealing well-focused tortuous vessels (*white arrow*). Original magnification ×10.

**Fig 3:**
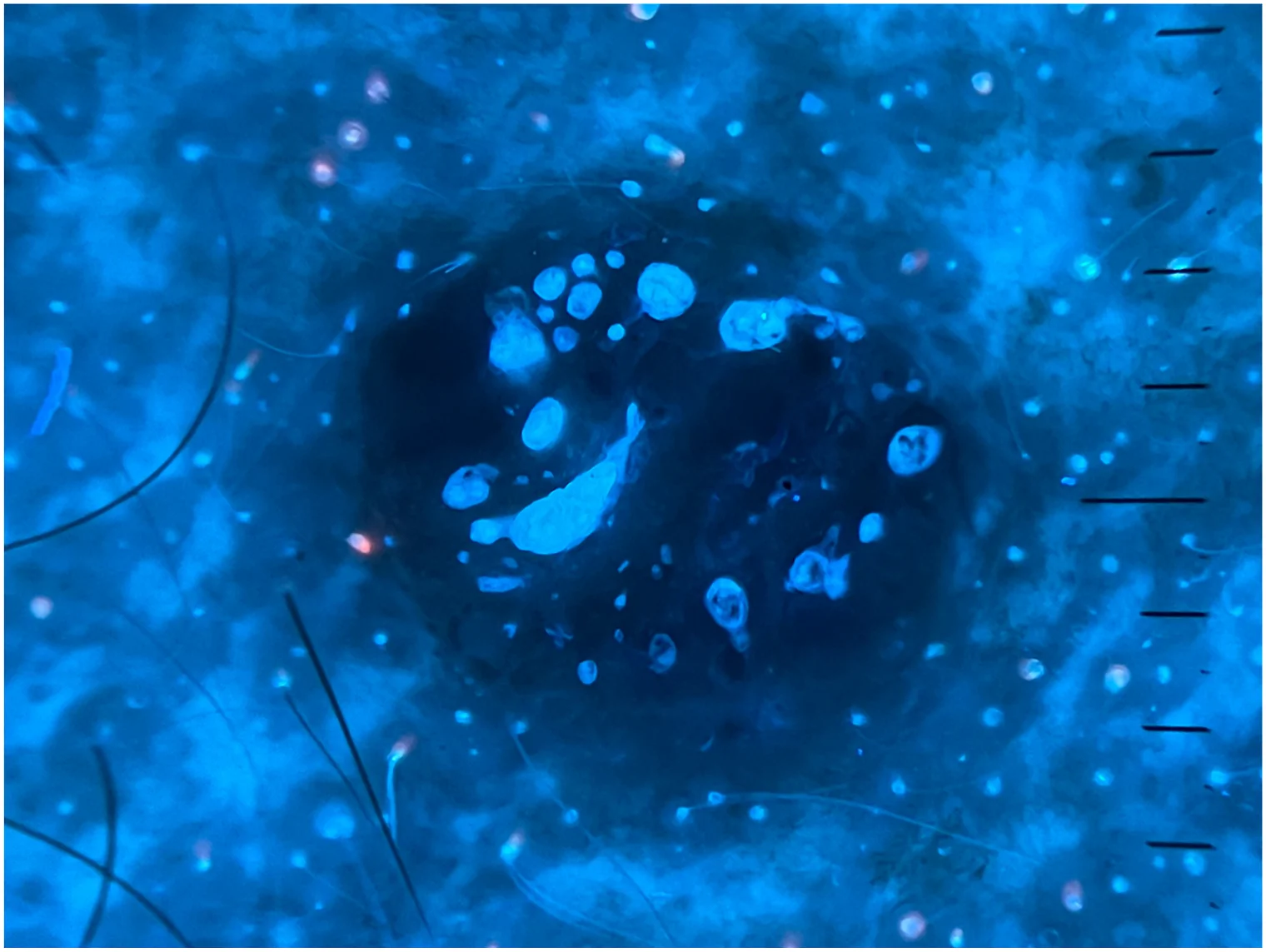
Ultraviolet-induced fluorescence dermoscopic image showing multiple blue-white fluorescent clods distributed throughout the lesion, corresponding to keratin-filled comedo-like openings. Original magnification ×10.

## Histologic diagnosis

Histopathologic examination revealed a basaloid epithelial neoplasm composed of solid nests connected to the epidermis and extending into the dermis, with peripheral palisading and stromal retraction clefts. Prominent lesional melanin pigmentation, keratin-filled epidermal invaginations, and cystic structures containing lamellated keratin were present. No intratumoral calcification or evidence of a coexisting seborrheic keratosis (SK) was identified. The diagnosis was pigmented basal cell carcinoma (BCC), predominantly nodular with a focal micronodular component (Fig 4).

Key message

Comedo-like openings and milia-like cysts are classic dermoscopic structures seen in SK and represent an unexpected finding in BCC. Although comedo-like openings have been described in the uncommon infundibulocystic variant, this case demonstrates that such SK-like dermoscopic features may also occur in other subtypes, coexisting with typical criteria of BCC. Importantly, characteristic BCC structures may occasionally be absent, making these features a diagnostic pitfall in pigmented lesions.^1^^,^^2^

**Fig 4:**
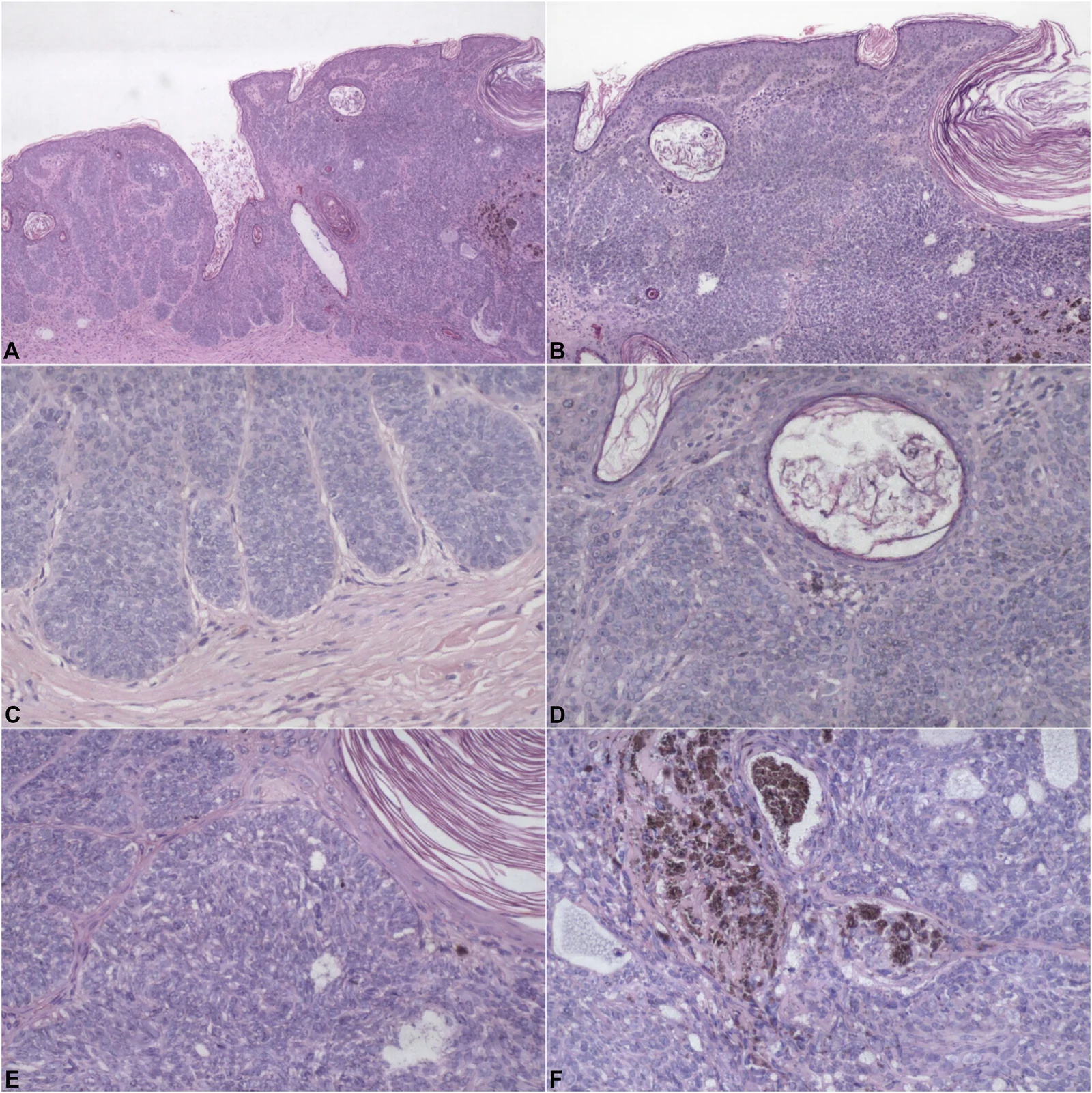
**A-F,** Hematoxylin and eosin–stained sections showing a basaloid epithelial neoplasm composed of solid tumor nests extending from the epidermis into the dermis, with peripheral palisading, stromal retraction clefts, keratin-filled epidermal invaginations and cystic structures, prominent intratumoral melanin pigmentation, and stromal melanophages.

### Declaration of generative AI and AI-assisted technologies in the writing process

None.

## Conflicts of interest

None disclosed.
